# Construal of self as a mental health inpatient: a systematic review and narrative synthesis of repertory grid studies

**DOI:** 10.3389/fpsyt.2025.1431798

**Published:** 2025-04-04

**Authors:** Eleanor Elizabeth Wozniak, Dougal Julian Hare, Lynsey Gregg, Anja Wittkowski

**Affiliations:** ^1^ Division of Psychology and Mental Health, School of Health Sciences, The University of Manchester, Manchester, United Kingdom; ^2^ Perinatal Mental Health and Parenting (PRIME) Research Unit, Greater Manchester Mental Health National Health Service (NHS) Foundation Trust, Manchester, United Kingdom; ^3^ Manchester Academic Health Science Centre, Manchester, United Kingdom

**Keywords:** adult mental health, psychiatric disorder, service user, repertory grid, personal construct theory, inpatient admission, construal of self

## Abstract

**Introduction:**

Mental health is influenced by how we perceive ourselves and others. A person’s conceptual structure and how he/she understands and makes sense of the world can be explored using the repertory grid technique (RGT), an assessment tool derived from personal construct theory. This review aimed to a) draw together relevant literature that had implemented the RGT to explore the conceptual system of a person diagnosed with a mental health condition necessitating psychiatric admission, b) synthesise research findings related to the structure and content of the conceptual system, and c) provide insights into how inpatient service users construed themselves and others to inform therapeutic practice.

**Methods:**

A systematic search of five electronic databases (MEDLINE, EMBASE, PsycINFO, CINAHL, and Web of Science) and thesis databases (EThOS and ProQuest), alongside manual searches in relevant articles and Google Scholar, was conducted. Included studies were appraised for methodological quality using the Quality Assessment Tool for Studies with Diverse Designs.

**Results:**

Twenty-one studies were identified and analysed using narrative synthesis. Of these 21 studies, 12 intentionally used a comparison group and compared the conceptual systems of people with different mental health diagnoses or compared conceptual systems of people with and without a diagnosed mental health condition. Findings from comparison group studies suggested that the self-esteem of a person diagnosed with a mental health condition was lower, compared to a person with no identified mental health diagnoses. Other people were typically idealised by people experiencing mental ill health; however, this finding was not observed in the experience of depression. Cognitive complexity, conceptual structure, and construing were variable across mental health conditions. Conceptual structures that were “simple” and characterised by “tight” construing were consistent with the profile of people with a mental health diagnosis, except for people with schizophrenia spectrum and psychotic disorders.

**Conclusions:**

The structure of a conceptual system differed in people with and without a mental health condition and across mental health diagnoses. Considerations for how the review findings could inform psychological therapy and suggestions for future research are offered.

## 1 Introduction

The World Health Organization (1) estimates that one in eight people is experiencing a mental health condition. Depression and anxiety disorders are the most common mental health conditions, with approximately 322 million and 264 million people living with these conditions, respectively (2). Mental health conditions are a pervasive public health concern. For example, approximately 14% of people in India (3), approximately 20% of people in Australia (4), and up to 25% of people in the UK (5) experience a mental health condition at some point in their lives. Depressive disorders are a major contributor to non-fatal loss in health and functioning worldwide (2). For example, in the UK and regions of the USA, depression is the single greatest cause of years lived with disability (2, 6). Indeed, in these two countries, the incidences of mental health conditions among children and adults are increasing (7–9).

Mental health conditions can be of such severity and/or significantly impact a person’s safety, wellbeing, and functioning that a person may require admission to an inpatient psychiatric hospital for care and treatment (10). Proportionately most people who experience a mental health condition can be safely supported in the community; however, for some, community-based services are deemed insufficient. For instance, approximately 3% of people in the UK required an inpatient psychiatric admission between 2020 and 2021 (11). Therefore, those admitted into inpatient psychiatric care are often people who present with the highest complexity and severity of mental ill health. It could be inferred that once a threshold is reached when inpatient psychiatric admission becomes necessary, a person’s mental health condition is notably more prominent or “active”, in comparison to someone residing in the community. It is imperative that people accessing inpatient psychiatric care receive purposeful admissions, inclusive of therapeutic, personalised, and timely assessment, intervention, and treatment (10). Without access to psychologically driven assessment and intervention, inpatient service users become increasingly vulnerable to longer inpatient stays (12). Prolonged inpatient psychiatric admissions have been shown to have detrimental consequences for inpatient service users including increased negative emotional states, an increased sense of shame, and worsening symptomology, such as depression, anxiety, and suicidality (13). Additionally, inpatient psychiatric stays are a high financial cost for healthcare systems; for instance, during the 2019/2020 financial year, £9 billion was spent on inpatient mental health care in the UK (14).

The nature of mental health conditions is complex and multifaceted. *Personal construct theory* (PCT) (15) is grounded in constructivist epistemology (16), which was presented with the term *constructive alternativism* (coined by Kelly, 15). This approach theorises that people construct their comprehension of the world and possess the capability to reconstruct their understanding following new experiences (17). The premise of PCT is that, through personal experience, people engage in a continuous process of forming subjective representations (i.e., “constructs”) of self-states (e.g., *ideal self*) and others (e.g., *mother* and *father*), which are referred to as “elements”. According to Kelly (15), a person uses his/her unique, and intricate, construct system to understand and interpret the world around him/her, a process termed *construing*, to make sense of and anticipate the behaviour of other people and thus sequentially his/her own behaviour. Kelly (18) postulates that a person’s personal construct system both enables and restricts his/her actions and has a pivotal role in shaping self-concept, an integral factor of our mental health. Kelly’s theory (15) also depicts the process by which construing occurs and posits that the nature of any person’s construing can be described along a “loose–tight” dimension. It is theorised that “loose” construing is when an element is positioned on opposing ends of construct poles on different occasions, resulting in changeable, vague predictions. The reverse pattern is the case in “tight” construing, which leads to consistent, unvarying, precise predictions (15). Kelly (15) further proposed that should a person construe extremely, either excessively “loosely” or “tightly”, it poses a risk to his/her psychological wellbeing.

Kelly (15) derived the complementary methodological approach of the repertory grid technique (RGT) from PCT. RGT is an assessment tool that allows for the exploration of a person’s views about the self and others through idiosyncratic language (19). A semi-structured interview is used to develop the repertory grid, and the procedural steps taken to form the grid can vary slightly. Typically, a person is asked to rate or rank various “elements” (e.g., *actual self* and *ideal self*; (20)) along several bipolar construct poles (e.g., “aggressive-easy going”; (21), p. 299) to determine the construct pole with which the person aligns the element more closely. Bipolar construct poles are often yielded from the person himself/herself through a method of elicitation carried out by the interviewer (e.g., triadic difference method; 15) and illustrated as numerical rating scales (e.g., 1–5 Likert scale; (22)).

The RGT yields both quantitative data, including the numerical rating given to an element along the bipolar construct pole, and qualitative data, such as elicited constructs. Consequently, both quantitative analytical approaches, for instance, principal component analysis (PCA; (23)), and qualitative analytical approaches, such as content analysis of elicited constructs [e.g (24).,], can be conducted (17, 25). A strength of RGT is that it facilitates the objective reporting and analysis of what is essentially subjective data (26) and therefore exceeds other methodological approaches with regard to minimising the risks of social desirability bias (27) and interviewer bias (28). Commonly, RGT analysis intends to reveal the degree of similarity construed between a particular pair of elements (e.g., by calculating the Euclidean distance between *actual self* and *ideal self*; (29)), the degree to which an element is construed as preferred or non-preferred (e.g., by calculating the mean ranked position of the *actual self* element along all construct poles within the repertory grid, with respect to the preferred pole (20);), and construing (e.g., by calculating the percentage of variance accounted for by the first principal component by conducting PCA; (30)). Therefore, by employing the RGT and appropriate analyses, a person’s self-concept, his/her self-esteem (e.g., discrepancy between *actual self* and *ideal self*), how he/she views himself/herself relative to others (e.g., discrepancy between *actual self* and *significant other*), and his/her construing (e.g., cognitive complexity, cognitive organisation, and cognitive articulation) can be deciphered.

Given that these aforementioned concepts can be deduced from an RGT, this illustrates the advantageous nature of this instrument in measuring important concepts in mental health. *Self-discrepancy theory* (31) offers one understanding of how a person can experience subjectively negative affect, a central facet of mental health conditions (32, 33). According to self-discrepancy theory, how a person views himself/herself, relative to other aspects of the self, has a formative and perpetuating role in his/her mental health. The theory postulates that emotional distress, across mental health diagnoses, can result from an incongruence perceived between a person’s *actual self*, *ideal self*, and *ought self* (34), with one’s self-esteem being negatively impacted when greater discrepancy between the *actual self* and *ideal self* arises (31). Self-esteem has been defined as a component of self-concept by Carl Rogers (35), a pioneer in the study of self-concept and self-esteem. For the purpose of this review, *self-concept* is defined as a person’s knowledge structure about himself/herself, who and what the self is (36), and self-esteem as an individual’s overall positive or negative evaluation of the self (37).

Low esteem has a causal role in the development of some mental health conditions, including depression and anxiety (38, 39), and in the development of complex feelings of sadness and disappointment (40). Hence, people with mental health conditions are found to experience a greater prevalence of discrepancies between the *actual self* and *ideal self* (34, 41), low self-esteem, and a negative view of the self (42, 43). Conversely, psychological wellbeing, in particular higher self-esteem, has been associated with greater congruence between *actual* and *ideal* self-concepts (44). Reducing the discrepancy between the *actual self* and *ideal self* has been shown to positively impact a person’s mental health (45, 46) and can be achieved through psychological interventions, including cognitive behavioural therapy and interpersonal psychotherapy (47).

A person’s self-concept, self-esteem, and mental health can also be influenced by the perception he/she holds about the *self* in relation to *others* and the response he/she receives from *others* towards the *self*. A process that is important to acknowledge when thinking about this is self-stigma, which occurs when a person experiencing a mental health condition accepts the prevailing negative attitudes, prejudices, and stereotypes about people with mental health conditions and then internalises or directs these towards himself/herself, resulting in a negative self-perception (48–50). Stereotypes held by the public about people with mental health conditions include being viewed as “dangerous”, “incompetent”, and “to be blamed” [(51), p. 343]. According to Link and Phelan (52), self-stigma can result in people who experience a mental health condition, perceiving themselves negatively or disadvantaged or even alienated, relative to other people. In their systematic review of 272 studies, Dubreucq et al. (53) noted that people who endorsed high levels of self-stigma were more likely to experience lower self-esteem and self-efficacy, greater psychiatric symptoms, higher sense of loneliness, poorer recovery, and poorer quality of life.

There is only one systematic review of studies that employed the RGT to explore the construing of self and others. In their systematic review of 15 studies focusing on psychosis only, García-Mieres et al. (54) found that people experiencing psychosis displayed high levels of *actual*–*ideal* and *self*–*significant other* discrepancies, fragmentation of self, and a rigid construct system. Although García-Mieres et al. (54) noted valuable insights into the content and construing demonstrated by people experiencing psychosis, the authors did not provide conclusions about the conceptual system beyond the experience of psychosis to other mental health conditions. Therefore, no systematic review to date has summarised, across mental health conditions, for people who require psychiatric hospitalisation, the content and process of self and other construing, captured by RGT. Through the identification, synthesis, and appraisal of existing RGT literature, this systematic review specifically aimed to address the question “How do people experiencing mental health conditions construe themselves and others, when the severity of their condition necessitates admission to a mental health hospital?”

## 2 Method

This systematic review and narrative synthesis were conducted in accordance with the Preferred Reporting Items for Systematic Reviews and Meta-Analyses (PRISMA) guidelines (55). The protocol for this review was registered with Prospero International Prospective Register of Systematic Reviews (Ref: CRD42024498543, https://www.crd.york.ac.uk/PROSPERO/display_record.php?RecordID=498543) in January 2024.

### 2.1 Information sources and search strategy

The search strategy was developed through consultation with the University of Manchester library services. Categories from the PICO tool (Population, Intervention, Comparison and Outcome (56); were used to guide the development of the search strategy. A search for relevant literature was conducted in five electronic databases—MEDLINE, EMBASE, PsycINFO, CINAHL, and Web of Science—February 2024. These databases were selected because they included published research related to mental health conditions: RGT and PCT. To ensure the identification of all relevant studies that met the inclusion criteria, additional searches were undertaken using ProQuest, EThOS, Google Scholar, and Google in February 2024, and the top 200 results on each platform were screened. Furthermore, reference lists of all included articles were also reviewed (57).

The search terms were informed by the titles, abstracts, and keywords of key reviews and papers. Initial pilot searches were completed to support the development of the final search strategy, which were categorised into “mental health patient”, “mental health inpatient hospital”, and “repertory grid technique” (see **Table 1**). In addition, Medical Subject Headings (MeSH) terms were used to identify synonyms and added as search terms to the relevant key search category. All the terms and concepts were combined with Boolean operators (“AND” and “OR”), and the search strategy implemented is illustrated in **Table 1**.

**Table 1:** Search terms by category, search strategy, and databases.

	Databases (platform)		
	PsycINFO (Ovid), Medline (Ovid), EMBASE (Ovid), CINAHL (EBSCOhost), and Web of Science (Clarivate)		
	Key search category	Search terms	MeSH terms identified from Ovid and EBSCOhost platforms
1	“Mental health patient”	(psychiatric OR mental health patient* OR mental health inpatient* OR psychiatric patient* OR psychiatric inpatient* OR service user* OR inpatient* OR hospital* patient* OR patient*)	(OR mental patient* OR mental illness patient*)
2	“Mental health inpatient hospital”	(mental health inpatient unit* OR mental health inpatient hospital* OR mental health hospital* OR psychiatric hospital* OR psychiatric unit* OR inpatient unit* OR clinic* OR hospital*)	(OR mental hospital* OR psychiatric department* OR mental institution* OR psychiatric ward*)
3	“Repertory grid technique”	(repertory grid technique OR repertory grid OR RGT OR grid technique* OR rep grid* OR repgrid* OR repertory technique* OR construal OR personal construct)	(OR Bannister repertory grid)
	Search strategy		
4	1 AND 3		
5	2 AND 3		
6	1 AND 2 AND 3		

MeSH, Medical Subject Headings.

### 2.2 Eligibility criteria

Studies had to meet the following inclusion criteria: 1) implementation of “standard” RGT as opposed to a derived version (e.g., Bannister–Fransella Grid Test of Thought Disorder); 2) 50% or above of the total sample being people presenting with a mental health condition as broadly defined by recognised diagnostic manuals [e.g., Diagnostic and Statistical Manual of Mental Disorders, fifth edition revised (DSM-5-TR), American Psychiatric Association (APA) (58), or well-established instruments as the diagnostic categories of schizophrenia spectrum and other psychotic disorders, bipolar and related disorders, depressive disorders, anxiety disorders, obsessive-compulsive and related disorders, trauma- and stressor-related disorders, dissociative disorders, and personality disorders, and were described within the study by diagnosis or reaching clinical cutoff scores on an appropriate clinical tool; 3) 50% or above of the total sample were people admitted to an inpatient mental health service; 4) construing was primarily with reference to themselves, themselves and another element (person), and/or the person’s internal experience or lived experience in relation to their mental health; 5) the study had to report specific examples of elements used but did not have to state each element individually; 6) the study discussed the content and/or structure of the person’s construct system and/or construing; 7) the text was written in or translated into English; 8) the study was published peer-reviewed research or grey literature, including doctoral dissertations/theses (59); and 9) the study reported original data.

Studies were excluded if 1) they were review papers and/or book chapters; 2) RGT was completed within a forensic setting due to their specialist nature; 3) RGT was completed with person(s) presenting with a condition that came under a broad diagnostic category *not* listed in the inclusion criteria (e.g., neurodevelopmental disorders, eating disorders, somatic symptoms and related disorders, and substance-related and addictive disorders) due to their specialist nature and use of unique elements, which were distinct to their studies; 4) RGT was primarily employed to explore the construal of person(s) presenting with a physical health condition, in which the physical health condition was the primary focus, to enable sole focus on the construal of person(s) experiencing a mental health condition; 5) RGT was used to derive another, idiosyncratic variable (e.g., therapy success); and 6) construing was primarily with reference to specific external aspects of their mental health or externalities in general (e.g., practical aspects of their mental health care such as observation levels, or externalities such as observable behaviour and death).

### 2.3 Screening and study selection procedure

Records identified from database searches were exported into the EndNote software (60), and any duplicate records were removed. The first author screened all remaining records by assessing the titles, keywords, and abstracts against the predefined inclusion and exclusion criteria. Any studies that did not meet the criteria were excluded. If eligibility was unclear, the record was retained at this stage. A second reviewer (IS) independently screened a sample of 10% (*n* = 1,046) of the total number of records identified from database searches (*n* = 10,467), with substantive agreement between the two reviewers (99.52%, kappa = 0.78), with any discrepancies being settled through discussion. The first author then conducted a full-text screening of all remaining articles and excluded studies that did not meet eligibility. Any disagreements were resolved in discussions with all authors. If a full text could not be obtained, it was removed. All remaining studies were included in the review.

### 2.4 Data extraction and analysis

Following the identification of articles, the first author completed data extraction into Microsoft Word. Extracted data were tabulated and included pertinent information, such as the study aims, demographic data of the sample, and main findings. A narrative synthesis (61) was performed to facilitate the structured extraction and synthesis of the findings across several studies to draw broad and robust conclusions on how people with mental health conditions that necessitated inpatient psychiatric admission construed themselves and others. The synthesis process was assessed for robustness through assessment and critical reflection of the methodological quality of the included studies and the analytical methods utilised within the synthesis (61).

Narrative synthesis enables the story of the literature to be told. It requires immersion in the literature, achieved through the process of reading and re-reading the included papers, to enable themes and patterns across the studies to emerge (61). Narrative synthesis permits the inclusion, comparison, and combining of heterogeneous quantitative, qualitative, and mixed-methods studies (62) and is useful to employ when the area of literature is underdeveloped (61).

### 2.5 Methodological quality assessment

The methodological quality of the studies was critically appraised using the Quality Assessment Tool for Studies with Diverse Designs (QATSDD (63)), a tool that evaluates studies with diverse designs, including qualitative, quantitative, or mixed methodological approaches. The QATSDD was deemed appropriate for the current review, as repertory grid methodology incorporates both qualitative and quantitative elements (64). As the QATSDD was originally developed for use in the discipline of psychology (65), it was easily modifiable to meet the aims of this review (64). It has been effectively employed in more than 80 reviews (65), and most specifically, it has been utilised successfully in the systematic review of repertory grid literature (66). The QATSDD was selected over other mixed- and multi-methods methodological quality assessments such as the Quality Assessment with Diverse Studies (QuADS (65)).

The QATSDD assesses 16 areas including consideration of the sample size and representativeness, description of the data collection procedure, justification for the analytical approach employed, and critical discussion of the strengths and limitations. Typically, all 16 items are applied to studies of mixed methodology, and some items are omitted when reviewing a study that adopts a purely quantitative or qualitative methodological approach. However, the QATSDD generates a percentage rating for each study, which enables comparison of all studies, irrespective of methodological approach (63). For this review, the following four items, “rationale for choice of data collection tool(s)”, “statistical assessment of reliability and validity of measurement tool(s)”, “fit between stated research question and method of data collection”, and “assessment of reliability of analytic process”, were omitted because they were not considered meaningful in a review of studies that all employed repertory grid methodology. For example, the reliability of the repertory grid analytical process is not assessed equivalently to other qualitative and quantitative methods (19).

Across the relevant 12 dimensions, each study was rated on a 4-point scoring scale: 3 (complete), 2 (moderately), 1 (very slightly), or 0 (not at all). Total scores were calculated and then presented as a percentage rating. A higher percentage rating implied stronger methodological quality (63). In accordance with other reviews that used the QATSDD (67, 68), methodological quality was categorised as poor (0%–24%), moderate (25%–49%), good (50%–75%), or high (76%–100%). To ensure the reliability of quality appraisal ratings, a second reviewer (IS) independently rated 10 of the identified papers (47.62%). Between raters, an agreement rating of 80.00% was achieved for overall quality rating, and an agreement rating of 70.83% was accomplished for identical individual domain ratings. Any discrepancies were resolved following discussion. No studies were excluded from the review based on quality.

## 3 Results

### 3.1 Search results

**Figure 1:**
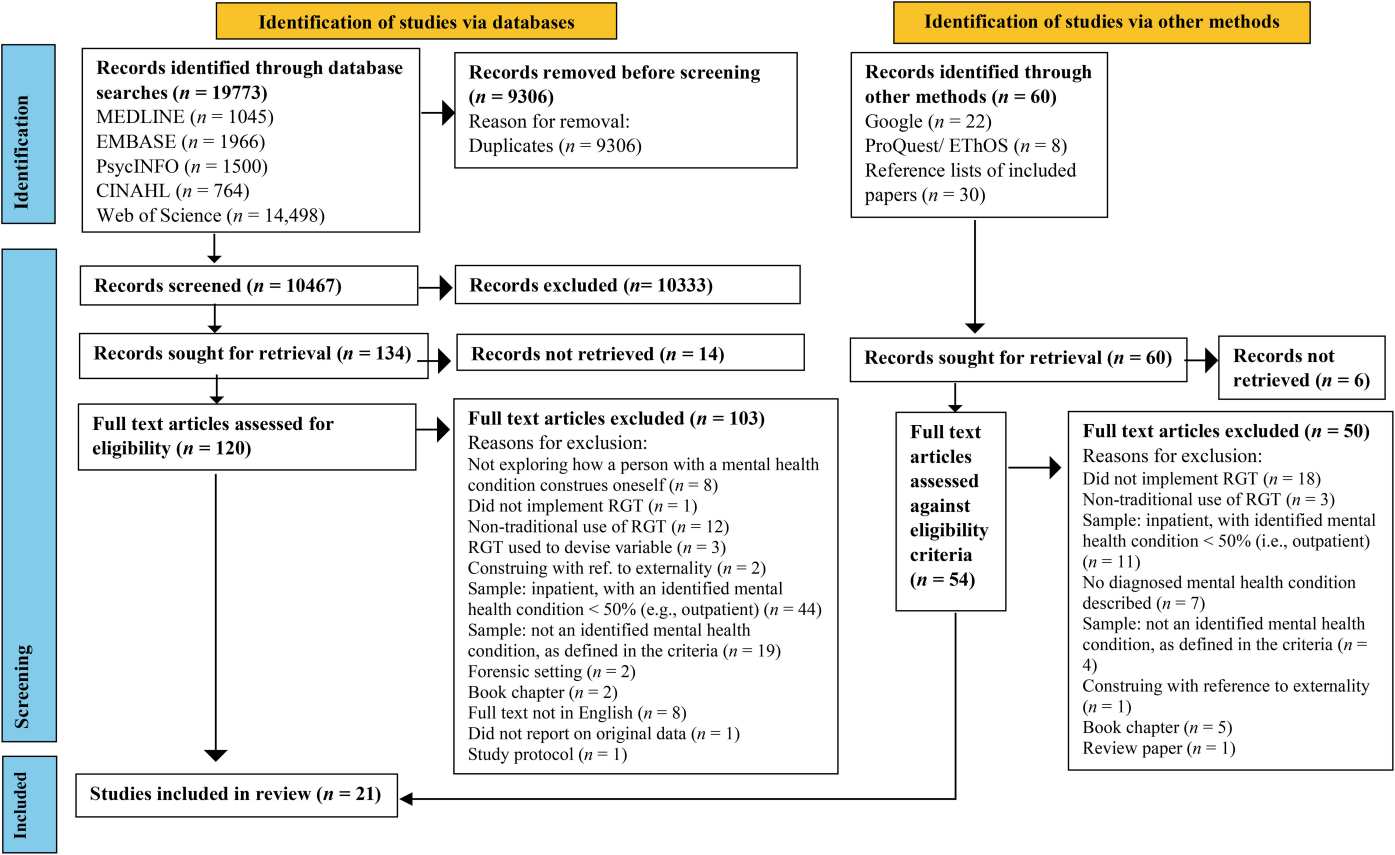
PRISMA flow diagram illustrating the review search strategy. PRISMA, Preferred Reporting Items for Systematic Reviews and Meta-Analyses.

A total of 19,833 records from database searches (*n* = 19,773) and other methods (*n* = 60) were initially identified (see **Figure 1**). After the removal of 9,306 duplicates, the titles and abstracts of the remaining 10,467 records were screened against eligibility criteria, and 10,333 were removed. Following database and additional method searches, 194 records were sought for retrieval, of which 20 were not obtained. A full-text review was undertaken of the remaining records (*n* = 174), of which 153 were excluded. Thus, 21 papers were included in the final review.

### 3.2 Study characteristics

The characteristics of the included studies are summarised in **Table 2**. Conducted between 1969 and 2014, studies originated from the UK (*n* = 9), the USA (*n* = 6), France (*n* = 2), and Germany (*n* = 4). Sample sizes ranged from N = 1 to N = 161 participants, with two studies (9.52%) implementing a single-case design (21, 72). All studies included a clinical group that comprised one or more participants with an identified mental health condition in an inpatient setting. In total, the studies collectively presented data from 579 participants with an identified mental health condition being supported in an inpatient setting, a further 25 participants who had an identified mental health condition but were residing in the community, and 156 participants with no identified mental health condition. Most studies reported about age (*n* = 18, 85.71%) and gender (*n* = 18, 85.71%). However, fewer studies reported on other socio-demographic factors including ethnicity (*n* = 3, 14.29%), employment status (*n* = 6, 28.57%), relationship status (*n* = 2, 9.52%), and education level (*n* = 6, 28.57%). The specific diagnoses of participants were detailed in 18 studies (85.71%).

**Table 2:** Demographic information and characteristics of the 21 included and reviewed studies.

	Study: authors, year, location	Sample size (n) and ratio	Diagnosis including validation of diagnosis	Demographic data	Setting
1	de Bonis et al. (1998) (69) France	Total (n = 47) Clinical group: unipolar MDD with BPD, inpatient (n = 17) Clinical group: MDD without BPD, inpatient (n = 12) Control group: no identified psychiatric condition, inpatient (n = 18)	Clinical group: unipolar MDD with BPD (n = 17); DSM-III-R Clinical group: MDD without BPD (n = 12), without BPD completely (n = 5), histrionic PD (n = 4), dependent PD (n = 3), PD not otherwise specified (n = 2); DSM-III-R Control group: no identified psychiatric condition (n = 18); NR	People with unipolar MDD with BPD, inpatient (clinical group): Age (years): mean = 30.23 (SD= 7.65), range = NR Gender: female (n = 15), male (n = 2) Ethnicity: NR, Employment: NR, Relationship status: NR Education level: high (n = 8), low (n = 9) Vocabulary score: mean = 25.17 (SD = 4.79) Severity of depression: mean= 10.29 (SD= 1.41) People with MDD without BPD, inpatient (clinical group): Age (years): mean = 38.58 (SD= 7.18), range = NR Gender: female (n = 9), male (n = 3) Ethnicity: NR, Employment: NR, Relationship status: NR Education level: mean = high (n = 5), low (n = 7) Vocabulary score: mean = 28.83 (SD = 7.37) Severity of depression: mean= 10.58 (SD= 3.14) People with no psychiatric condition, inpatient (control group): Age (years): mean = 29.50 (SD= 9.17), range = NR Gender: female (n = 10), male (n = 8) Ethnicity: NR, Employment: hospital staff (n = NR), medical school administrative staff (n = NR), medical students (n = NR), Relationship status: NR Education level: mean = high (n = 12), low (n = 6) Vocabulary score: mean = 29.14 (SD = 3.08) Severity of depression: mean= 1.11 (SD= 1.41)	Clinical groups: inpatient psychiatric unit, the Bicetre University Hospital Control group: NR
2	Axford and Jerrom (1986) (20) UK	Total (n = 30) Clinical group: depression, inpatient (n = 10) Control group: other identified psychiatric condition, inpatient (n = 10) Control group: no identified psychiatric condition, inpatient (n = 10)	Clinical group: major unipolar depression (n = 10); diagnosed by psychiatrist and BDI clinical cut off Psychiatric control group: anxiety or PD (n = 10); diagnosed by psychiatrist and BDI clinical cut off Control group: acute medical conditions (n = 10); BDI clinical cut off	Clinical group: Age (years): mean = 40.0 (SD= NR), range = NR Gender: female (n = 5), male (n = 5) Ethnicity: NR, Employment: NR, Relationship status: NR, Education level: NR Control group (psychiatric): Age (years): mean = 51.0 (SD= NR), range = NR Gender: female (n = 5), male (n = 5) Ethnicity: NR, Employment: NR, Relationship status: NR, Education level: NR Control group (non-psychiatric): Age (years): mean = 55.0 (SD= NR), range = NR Gender: female (n = 5), male (n = 5) Ethnicity: NR, Employment: NR, Relationship status: NR, Education level: NR	Clinical group: Bellsdyke Hospital, an inpatient unit providing longer term care and treatment for severe and enduring mental health problems Control group: medical wards of general hospital
3	Ashworth et al. (1982) (30) UK	Total (n = 70) Clinical groups: (n = 2) an identified psychiatric condition, inpatient (n = 30) Control groups: (n = 2) an identified psychiatric condition, inpatient (n = 20) Control group: (n = 1) an identified psychiatric condition, community (n = 10) Control group: (n = 1) physical illness and no identified psychiatric condition, inpatient (n = 10)	Clinical groups: depression (n = 20); NR, mania (n = 10); diagnosed by psychiatrist Control groups (psychiatric inpatient): schizophrenia (n = 10), alcoholism (n = 10); diagnosed by psychiatrist Control group (psychiatric community): recovered depression (n = 10); diagnosed by psychiatrist Control group (non-psychiatric inpatient): physical illness (n = 10); diagnosed by medical staff	People with depression, inpatient (clinical group): Age (years): mean = 47.6 (SD= 12.3), range = 24-68 Gender: female (n = 11), male (n = 9) Ethnicity: NR, Employment: NR, Relationship status: NR, Education level: NR MHIQE: mean= 107.1 (SD=14.9), range = 83-130 People with mania, inpatient (clinical group): Age (years): mean = 42.7 (SD= 14.9), range = 22-61 Gender: female (n = 3), male (n = 7) Ethnicity: NR, Employment: NR, Relationship status: NR, Education level: NR MHIQE: mean= 107.6 (SD=13.9), range= 90-130 People with schizophrenia, inpatient (control group): Age (years): mean = 35.1 (SD= 10.9), range = 19-58 Gender: female (n = 3), male (n = 7) Ethnicity: NR, Employment: NR, Relationship status: NR, Education level: NR MHIQE: mean= 99.7 (SD=7.6), range= 85-106 People with alcoholism, inpatient (control group): Age (years): mean = 38.4 (SD= 8.9), range = 26-48 Gender: female (n = 1), male (n = 9) Ethnicity: NR, Employment: NR, Relationship status: NR, Education level: NR MHIQE: mean = 101.1 (SD=9.4), range = 92-118 People recovered from depression, community (control group): Age (years): mean = 45.1 (SD= 10.8), range = 26-56 Gender: female (n = 4), male (n = 6) Ethnicity: NR, Employment: NR, Relationship status: NR, Education level: NR MHIQE: mean = 113.3 (SD=12.6), range = 92-128 People with a physical illness, inpatient (control group): Age (years): mean = 45.6 (SD= 10.1), range = 31-59 Gender: female (n = 5), male (n = 5) Ethnicity: NR, Employment: NR, Relationship status: NR, Education level: NR MHIQE: mean = 103.0 (SD=7.9), range = 89-114	Clinical groups: a Medical Research Council research unit Control group (schizophrenia, inpatient): NR Control group (alcoholism, inpatient): alcoholism treatment unit Control group (psychiatric, community): NR Control group (non-psychiatric, inpatient): general medical and surgical wards
4	Hewstone et al. (1981) (70) UK	Total (n = 20) Clinical group: an identified psychiatric condition, inpatient (n = 10) Control group: no identified psychiatric condition, inpatient (n = 10)	Clinical group: neurotic depression (n = 10); NR Control group: no identified psychiatric condition (n = 10); NR	Clinical group: Age (years): mean = 45.3 (SD= NR), range = 28-71 Gender: female (n = 10), male (n = 0) Ethnicity: NR, Employment: NR, Relationship status: NR, Education level: NR Control group: Age (years): matched to clinical group Gender: female (n = 10), male (n = 0) Ethnicity: NR, Employment: NR, Relationship status: NR, Education level: NR	Clinical group: Sarah Britton Wills Unit (inpatient psychiatric unit) of the Bristol General Hospital Control group: University department of medicine from the Bristol Royal Infirmary
5	Space and Cromwell (1980) (71) USA	Total (n = 57) Clinical group: depression, inpatient (n = 19) Control group: other identified psychiatric condition, inpatient (n = 19) Control group: no identified psychiatric condition, community (n = 19)	Clinical group: manic depressive, depressed type (n = 3), involutional melancholia (n = 1), psychotic depression, unipolar (n = 1), neurotic/reactive depression (n = 14); DSM-II and BDI Control group (psychiatric): hysterical personalities (n = 4), conversion hysteria (n = 1), inadequate personalities (n = 1), schizoid personality (n = 1), anxiety neuroses (n = 3), passive dependent personalities (n = 2), marriage maladjustments (n = 2), adjustment reactions to adult life (n = 2), phobic neuroses (n = 1), mixed PD (n = 2); DSM-II and BDI Control group (non-psychiatric): no identified psychiatric condition (n = 19); BDI	People with depression, inpatient (clinical group): Age (years): mean = 37.16 (SD= 12.47), range = 19-57 Gender: female (n = 14), male (n = 5) Ethnicity: NR, Employment: NR, Relationship status: NR Education level: mean = 12.05 years (SD= 1.93), range= 7-14 years Peabody Picture Vocabulary Test: mean= 123.51 (SD= 8.91), range= 111-141 People with other psychiatric condition, inpatient (control group): Age (years): mean = 35.32 (SD= 10.31), range = 21-57 Gender: female (n = 14), male (n = 5) Ethnicity: NR, Employment: NR, Relationship status: NR Education level: mean = 12.11 years (SD= 1.80), range = 8-15 years Peabody Picture Vocabulary Test: mean = 124.26 (SD= 11.32), range = 107-141 People with no identified psychiatric condition, community (control group): Age (years): mean = 34.21 (SD= 14.38), range = 19-63 Gender: female (n = 14), male (n = 5) Ethnicity: NR Employment: employees at the hospitals (n = 19) Relationship status: NR Education level: mean = 12.68 years (SD= 1.22), range = 11-16 years Peabody Picture Vocabulary Test: mean = 125.42 (SD= 11.65), range = 102-142	Clinical group: neuropsychiatric hospital Control group (psychiatric): neuropsychiatric hospital Control group (non-psychiatric): employees at neuropsychiatric hospital
6	Rowe (1971) (21) UK	Total (n = 1) Inpatient (n = 1) Community (n = 0)	Recurrent depression (n = 1); NR	Age (years): 38 Gender: female (n = 1), male (n = 0) Ethnicity: NR Employment: Office worker (n = 1) Relationship status: married (n = 1) Education level: left school at 14 (n = 1)	Hospital (details NR)
7	Rowe (1969) (72) UK	Total (n = 1) Inpatient (n = 1) Community (n = 0)	Recurrent depression (n = 1); NR	Age (years): 63 Gender: female (n = 1), male (n = 0) Ethnicity: NR, Employment: NR, Relationship status: NR, Education level: NR	Hospital (details NR)
8	Catina and Tschuschke (1993) (73) Germany	Total (n = 15) Inpatient (n = 15) Community (n = 0)	Neuroses (n = 15); DSM-III	Age (years): mean = NR (SD= NR), range = NR Gender: female (n = 10), male (n = 5) Ethnicity: NR, Employment: NR, Relationship status: NR, Education level: NR	the Psychotherapeutic Clinic Sonnenberg in Stuttgart
9	Millar (1980) (74) UK	Total (n = 30) Clinical group: an identified psychiatric condition, inpatient (n = 15) Control group: no identified psychiatric condition matched sample, community (n = 15)	Clinical group: OCD (n = 15); LOI Control group: no identified psychiatric condition (n = 15); LOI	Clinical group: Age (years): mean = 30.1 (SD= NR), range = NR Gender: female (n = 12), male (n = 3) Ethnicity: NR, Employment: NR, Relationship status: NR, Education level: NR Control group: Age (years): mean = 29.6 (SD= NR), range = NR Gender: female (n = 12), male (n = 3) Ethnicity: NR Employment: nursing staff (n = 15) Relationship status: NR, Education level: NR	Clinical group: St Lukes-Woodside hospital Control group: NR
10	Makhlouf-Norris and Norris (1972) (75) UK	Total (n = 22) Clinical group: an identified psychiatric condition, inpatient (n = 11) Control group: no identified psychiatric condition, inpatient (n = 11)	Clinical group: OCD (n = 11); NR Control group: no identified psychiatric condition (n = 11); NR	Clinical group: Age (years): mean = NR (SD= NR), range = NR Gender: NR Ethnicity: NR, Employment: NR, Relationship status: NR, Education level: NR Control group: Age (years): mean = NR (SD= NR), range = NR Gender: NR Ethnicity: NR, Employment: NR, Relationship status: NR, Education level: NR	Clinical group: hospital (details NR) Control group: hospital (details NR)
11	Makhlouf-Norris et al. (1970) (76) UK	Total (n = 22) Clinical group: an identified psychiatric condition, inpatient (n = 11) Control group: no identified psychiatric condition, inpatient (n = 11)	Clinical group: OCD (n = 11); NR Control group: no identified psychiatric condition (n = 11); NR	Clinical group: Age (years): mean = NR (SD= NR), range = NR Gender: NR, Ethnicity: NR, Employment: NR, Relationship status: NR, Education level: NR Control group: Age (years): mean = NR (SD= NR), range = NR Gender: NR, Ethnicity: NR, Employment: NR, Relationship status: NR, Education level: NR	Clinical group: hospital (details NR) Control group: hospital (details NR)
12	Paget and Ellett (2014) (22) UK	Total (n = 30) Inpatient (n = 15) Community (n = 15)	Psychotic illness, with a current persecutory belief: paranoid schizophrenia (n = 23), schizoaffective disorder (n = 2), residual schizophrenia (n =1), delusional disorder (n = 2), schizophrenia (n = 2); diagnosed by psychiatrist and SAPS rating [89]	Age (years): mean = 36.4 (SD= 11.1), range = 23-64 Gender: female (n = 12), male (n = 18) Ethnicity: White (n = 16), Black (n = 3), Asian (n = 8), Other (n = 3) Employment: unemployed (n = 27), long-term sick leave (n = 2), unknown (n = 1) Relationship status: NR Education level: up to secondary school (n = 16), further education (n = 8), higher education (n = 6) Length of illness (years): mean = 11.6 (SD= 8.53), range = NR Number of psychotic episodes: mean = 4 (SD = 2.09), range = 2-10	Inpatient and outpatient services in London NHS Foundation Trusts (n = 2)
13	Böker et al. (2000) (29) Germany	Total (n =161) Clinical group: an identified psychiatric condition, inpatient (n = 127) Control group: no identified psychiatric condition, inpatient (n = 34)	Clinical group: BAD (n = 10), BAD in remission (n = 30), recurrent affective disorder in remission (n = 29), dysthymia (n = 30), schizoaffective disorders (n = 28); ICD-10 Control group: orthopaedic–surgical patients (n = 34); NR	Clinical group: Age (years): mean = 42.2 (SD= 12.1), range = NR Gender: female (n = 75), male (n = 52) Ethnicity: NR, Employment: NR, Relationship status: NR, Education level: NR Control group: Age (years): mean = 43.9 (SD= 13.0), range = NR Gender: female (n = 18), male (n = 16) Ethnicity: NR, Employment: NR, Relationship status: NR, Education level: NR	Clinical group: the Psychiatric University hospital of Frankfurt Control group: the Orthopaedic University Hospital of Frankfurt
14	Adelman (1998) (77) USA	Total (n = 9) Inpatient (n = 9) Community (n = 0)	Schizophrenia (n = 5), schizoaffective disorder (n = 4); NR	Age (years): mean = 44.3 (SD= NR), range = 25-56 Gender: female (n = NR), male (n = NR) Ethnicity: NR, Employment: NR, Relationship status: NR, Education level: NR Estimated IQ: above average (n = 2), average (n = 4), below average (n = 2), borderline (n = 1) Number of hospital admissions: mean = 3.4, range = 1-7	A state psychiatric hospital
15	Kubiak (1998) (78) USA	Total (n =33) Inpatient (n = 33) Community (n = 0)	Schizophrenia (n = 29), BAD (n = 2), recurrent MDD (n = 1), adjustment disorder with mixed disturbance of emotions and conduct (n = 1); NR	Administration 1 (n = 6): Age (years): 35.5 (SD = 7.66), range = NR Gender: female (n = 2), male (n = 4) Ethnicity: White (n = 4), Black (n = 2) Employment: NR Relationship status: married (n = 1), never married (n = 5) Education level: NR Days from admission to ad. 1: 310.83 (SD= 195.82) No. of previous hospitalisations: 6.0 (SD= 4.15) Administration 2 (n = 10): Age (years): 38.10 (SD = 10.59), range = NR Gender: female (n = 2), male (n = 8) Ethnicity: White (n = 9), Black (n = 1) Employment: NR Relationship status: married (n = 2), divorced (n = 4), never married (n = 4) Education level: NR Days from admission to ad. 1: 166.10 (SD= 210.29) No. of previous hospitalisations: 5.90 (SD= 3.14) Administration 3 (n = 17): Age (years): 36.0 (SD = 9.74), range = NR Gender: female (n = 10), male (n = 7) Ethnicity: White (n = 15), Black (n = 1), Hispanic (n = 1) Employment: NR Relationship status: married (n = 3), divorced (n = 4), never married (n = 9), widowed (n = 1) Education level: NR Days from admission to ad. 1: 225.18 (SD= 197.07) No. of previous hospitalisations: 5.47 (SD= 2.21)	A long-term rehabilitation program located at the Lincoln Regional Center, a psychiatric hospital operated by the State of Nebraska
16	de Bonis et al. (1995) (79) France	Total (n = 54) Clinical group: schizophrenia, inpatient (n = 19) Clinical group: BPD, inpatient (n = 17) Control group: no identified psychiatric condition, inpatient (n = 18)	Clinical group: paranoid schizophrenia (n = 10), disorganised schizophrenia (n = 4), undifferentiated schizophrenia (n = 4), residual schizophrenia (n = 1); DSM-III-R Clinical group: BPD (n = 17); DSM-III-R Control group: no identified psychiatric condition (n = 18); NR	People with schizophrenia, inpatient (clinical group): Age (years): mean = 29.63 (SD= 16.83), range = NR Gender: female (n = 10), male (n = 9) Ethnicity: NR, Employment: NR, Relationship status: NR Education level: high (n = 8), low (n = 11) Vocabulary score: mean = 24.37 (SD= 15.45) Severity of depression: mean= 7.32 (SD= 4.67) People with BPD, inpatient (clinical group): Age (years): mean = 30.23 (SD= 7.65), range = NR Gender: female (n = 15), male (n = 2) Ethnicity: NR, Employment: NR, Relationship status: NR Education level: mean = high (n = 8), low (n = 9) Vocabulary score: mean = 25.17 (SD= 4.79) Severity of depression: mean= 10.29 (SD= 4.36) People with no psychiatric condition, inpatient (control group): Age (years): mean = 29.50 (SD= 9.17), range = NR Gender: female (n = 10), male (n = 8) Ethnicity: NR Employment: hospital staff (n = NR), medical school administrative staff (n = NR), medical students (n = NR) Relationship status: NR Education level: mean = high (n = 12), low (n = 6) Vocabulary score: mean = 29.14 (SD= 3.08) Severity of depression: mean= 1.11 (SD= 1.41)	Clinical groups: inpatient psychiatric unit, the Bicetre University Hospital Control group: NR
17	Klion (1988) (80) USA	Total (n = 40) Clinical group: schizophrenia, Inpatient (n = 20) Control group: other identified psychiatric condition, inpatient (n = 20)	Clinical group: schizophrenia paranoid type (n = 10), schizophrenia undifferentiated type (n = 5), schizoaffective disorder (n = 4), schizophrenia residual type (n = 1); DSM-III Control group: major depression (n = 5), adjustment reaction (n = 5), BAD (n = 3), dysthymic disorder (n = 3), brief reactive psychosis (n = 2), PD (n = 2); DSM-III	Clinical group: Age (years): 31.3 (SD= NR), range = NR Gender: female (n = NR), male (n = NR) Ethnicity: NR, Employment: NR, Relationship status: NR, Education level: NR Previous hospitalisation: at least one admission documented (90%) Control group: Age (years): 29.2 (SD= NR), range = NR Gender: female (n = NR), male (n = NR) Ethnicity: NR, Employment: NR, Relationship status: NR, Education level: NR Previous hospitalisation: at least one admission documented (75%)	Clinical group: short-term, state-run psychiatric inpatient hospital Control group: short-term, state-run psychiatric inpatient hospital
18	Krauthauser et al. (1994) (81) Germany	Total (n =141) Clinical group: subsample who completed repertory grids, inpatient (n = 22) Control group: randomly generated computer program grids (n = 20)	Clinical group: anxiety disorder (n = NR), chronic psychogenic or psychosomatic pain syndrome (n = NR), depression (n = NR), hysteria (n= NR); DSM-III-R	Total sample: Age (years): mean = 34.4 (SD= 9.7), range = 17-56 Gender: female (n = 80), male (n = 61) Ethnicity: NR, Employment: NR, Relationship status: NR, Education level: NR Sub sample: Age (years): mean = NR (SD= NR), range = NR Gender: female (n = 13), male (n = 9) Ethnicity: NR, Employment: NR, Relationship status: NR, Education level: NR	the Mainz University Hospital
19	Tschuschke and Dies (1994) (82) Germany	Total (n = 16) Inpatients (n = 16) Community (n = 0)	Presentations of most participants were “axis II characterological problems and comorbid anxious and/or depressive symptomatology” (p. 188); DSM-III-R	Age (years): 29.0 (SD= NR), range = NR Gender: female (n = 9), male (n = 7) Ethnicity: NR, Employment: NR, Relationship status: NR, Education level: NR	Hospital (details NR)
20	Phillips (1981) (83) USA	Total (n = 60) Inpatient (n = 60) Community (n = 0)	Psychiatric inpatients (n = 60); NR	Age (years): 27.0 (SD= NR), range = 16 - 51 Gender: female (n = 32), male (n = 28) Ethnicity: White (n = 60) Employment: NR, Relationship status: NR, Education level: NR	Private psychiatric hospital in New England
21	Phillips (1976) (84) USA	Total (n = 20) Inpatient (n = 20) Community (n = 0)	Presentations “ranged from moderate reactive depressions to presentation of psychotic symptoms” (p. 952); NR	Age (years): 41.0 (SD= NR), range = 20 – 65 Gender: female (n = 0), male (n = 20) Ethnicity: NR, Employment: NR, Relationship status: NR Education level: “ranged from failure to complete elementary education to completion of the baccalaureate degree” (p. 952)	Regional veterans’ administration centre

NR, not reported; OCD, obsessive compulsive disorder; BAD, bipolar affective disorder; MDD, major depressive disorder; PD, personality disorder; BPD, borderline personality disorder; MHIQE, Mill Hill IQ Equivalent; SAPS, Scale for the Assessment of Positive Symptoms (85); LOI, Leyton Obsessional Inventory (86); BDI, Beck Depression Inventory (87); PCP = personal construct psychology; RG, repertory grid; ICD-10, the International Classification of Diseases 10th edition (88); DSM, Diagnostic and Statistical Manual of mental disorders (89–91).

Across 21 studies, participants in the clinical groups were predominately diagnosed with a depressive disorder (*n* = 7), a schizophrenia spectrum and other psychotic disorders (*n* = 6), or an anxiety disorder (*n* = 4). Specific diagnoses were unclear in four studies (19.05%): examples of diagnoses were provided, or diagnoses were generalised and reported as a psychiatric condition. A validation of diagnosis method was reported in 12 studies (57.14%), such as being diagnosed by the treating clinician (*n* = 3), the International Classification of Diseases, 10th edition (ICD-10; WHO (88); *n* = 1), or an edition of the DSM (*n* = 1, DSM-II, APA, 71; *n* = 2, DSM-III, APA (89); *n* = 4, DSM-III-R, APA, 73) or by reaching clinical cutoff on an appropriate questionnaire or measure (*n* = 4).

Regarding the implementation of the RGT, all studies provided a pre-determined list of elements to the participants. In 18 studies (85.71%), idiosyncratic constructs were elicited from participants through a range of methods. In three studies (14.29%), participants were provided with at least one construct, and two studies (9.52%) failed to specify whether constructs were elicited or pre-determined. The repertory grid was implemented on a single occasion for each participant in 16 studies (76.19%), while five studies (23.81%) repeated the application of the repertory grid at least once. Twelve studies (57.14%) utilised at least one comparison group.

### 3.3 Methodological quality of included studies

Overall, the methodological quality of the 21 studies was considered to be good (*n* = 17) or moderate (*n* = 3), with one study rated as being high quality ((22); see **Supplementary Table 1**). Irrespective of the rating, limitations were identified across all studies. However, given the scarcity of relevant studies in this research field, the collective perspective among the authors was that all included studies would meaningfully contribute to the review question, and hence, no studies were excluded on the grounds of methodological rigour.

Studies rated as moderate (21, 72, 77) lacked thorough consideration or description of various aspects of research design, including an explicit statement of aims, research setting, recruitment data, sample size and analysis, sample representativeness, data collection procedure, justification of analysis method, service user involvement, and strengths and limitations. However, it should be noted that some authors seemed to observe limited word counts, which resulted in papers lacking adequate detail (21, 72, 84).

Overall, 16 studies (76.19%) scored highly in relation to providing an explicit statement concerning the theoretical framework, and 14 studies (66.67%) were rated “complete” regarding an explicit statement of aims/objectives. Two studies [ (21, 79); 9.52%] failed to be assigned the highest rating on “fit between research question and format and content of data collection tool”. One of the 21 studies was rated below high on “fit between research question and method of analysis” [(72); 4.76%]. Conversely, 10 studies (47.62%) did not critically discuss their strengths and limitations, and 17 studies (80.95%) failed to provide any detailed recruitment data. No study demonstrated evidence of service user involvement in study design, and only one study had reported consideration of sample size [(22); 4.76%].

### 3.4 Synthesis of repertory grid technique findings

#### 3.4.1 Implementation of a comparison group

As previously mentioned, 12 studies (57.14%) utilised at least one comparison group, while nine studies (42.86%) did not (see **Table 2**). Studies that did not implement a comparison group presented information on the conceptual systems of specific mental health diagnoses in isolation. Conversely, studies that implemented a comparison group enabled authors to draw comparisons between the conceptual systems of people with and without a mental health condition and/or between different mental health diagnoses. The method of comparison varied across the literature, with most authors performing statistical comparisons between groups [e.g., t-tests (20)]. Alternatively, other authors presented exploratory comparisons to illustrate similarities and differences in construals and construing across groups [e.g (74)]. Therefore, this review was able to synthesise findings relating to conceptual systems and group these according to specific diagnoses and, when possible, summarise the comparisons made across different diagnoses as well as between people with and without mental health conditions.

#### 3.4.2 Construal in relation to self

A central concept was investigated in the 21 included studies related to the self-esteem and construal of self commonly experienced by a person diagnosed with a mental health condition accessing inpatient psychiatric care (see **Supplementary Table 2**). Across studies, self-esteem was predominantly represented by the *actual–ideal* discrepancy, with greater discrepancy indicative of lower self-esteem [e.g (30)]. Construal of self was defined in several ways, including by the mean ranked position of the *actual self* element along all construct poles within the repertory grid, with respect to the preferred pole [e.g., (20)]. For example, if the *actual self* element had a lower mean ranked position along construct poles, it suggested that this element more closely applied to the preferred ends of the construct poles; therefore, a more positive construal of self was held (20).

##### 3.4.2.1 Self-esteem

For people experiencing a mental health condition, irrespective of diagnoses, reduced self-esteem was associated with increasing duration of mental health condition (29).

Specifically, low self-esteem was observed to be characteristic of depression (20, 30, 70–72). People diagnosed with depression appeared to experience lower self-esteem compared to people with no identified mental health condition and people diagnosed with other mental health conditions including anxiety disorders, personality disorders (PD), and mania, as illustrated in comparison group studies (20, 30, 70). One contrary to this finding was Böker et al. (29), who did not find a significant difference in self-esteem between people diagnosed with depression and people with no identified mental health condition, although dysthymic mood did tend to be associated with lower self-esteem.

Overall, the RGT findings relating to self-esteem and anxiety disorders were inconclusive. Catina and Tschuschke (73) assessed changes to self-esteem following therapeutic intervention for participants diagnosed with an anxiety disorder and found a greater *actual*–*ideal* discrepancy was observed in people who continued to present with heightened anxiety relative to those with reduced anxiety symptomology. This finding could have implied that lower self-esteem was associated with experiencing increased anxiety symptomology in anxiety disorders. However, Axford and Jerrom (20) showed that self-esteem was comparable between people with an anxiety disorder and people with no identified mental health condition, thus suggesting that the presence of anxiety symptomology may not necessarily coincide with lower self-esteem.

Interestingly, participants with an anxiety disorder were revealed to experience higher self-esteem in comparison to participants diagnosed with obsessive-compulsive disorder (OCD; (74)). However, Millar (74) did suggest that the difference in self-esteem between participants with OCD and anxiety disorders could possibly relate to the severity of the condition as opposed to the specific condition itself, and hence this finding should be interpreted cautiously. The use of RGT by authors led to the discovery that OCD was characterised by low self-esteem (74, 75) and by perceiving different self-states (i.e., *actual* and *social*) as non-ideal (75). Self-esteem was observed to be lower for people diagnosed with OCD, relative to people without a mental health condition, by authors who conducted comparison group studies (74, 75). Therefore, it seems that this review was able to make firmer conclusions concerning lower self-esteem appearing in OCD than it was in the case of anxiety disorders.

The RGT enabled authors to reveal that low self-esteem appeared to be a characteristic of bipolar affective disorder (29) and schizophrenia (30). RGT findings showed self-esteem to improve as psychological stabilisation occurred in bipolar affective disorder, which indicated a relationship between lower self-esteem and episodes of de-stabilisation in this disorder (29). However, one comparison group study did not show a significant difference in a measure of self-esteem between people with schizoaffective disorders and people with no identified mental health condition (29). Furthermore, a different comparison group study highlighted higher self-esteem in people experiencing mania, a central characteristic of bipolar affective disorder (92), compared to people with no identified mental health condition (30). These latter findings hindered the review’s ability to produce robust conclusions about how self-esteem may present in relation to distinct aspects of psychotic disorders and related experiences.

##### 3.4.2.2 Construal of self

Of the studies that provided findings relating to construal of self, all but one were conducted with people diagnosed with depression. Several studies revealed that depression was characterised by a negative construal of self (20, 69, 71, 72). In fact, de Bonis et al. (69) tentatively speculated that depressive mood was in fact responsible for negative evaluative self-content. However, Rowe (21) challenged this dominant connection between depression and a negative construal of self by suggesting that depression was perceived positively by the person experiencing it and the person diagnosed with depression perceived himself/herself to possess attributes that he/she valued, for example, being “generous”, “soft”, and “affectionate” (p. 298). Nonetheless, it must be acknowledged that the latter study was a single case design study; therefore, it may not be possible to generalise these results to wider experiences of depression (93).

In contrast, an alternative explanation was that, in depression, the *actual self* is construed both negatively and positively along interrelated dimensions (71, 94) as opposed to being construed consistently negatively (20, 69, 72). Space and Cromwell’s (73) perspective suggested that depression is characterised by an impeded ability to hold a secure, consistent construal of self, which differs from conclusions made by other authors that depression is associated with holding a consistent, albeit negative evaluation of self (20, 69, 72). In one study by Millar (74), OCD was found to be characterised by an extremely negative construal of self.

#### 3.4.3 Construal in relation to others

Several studies explored how people with mental health conditions construed other people and how they construed the self in relation to other people. Studies frequently captured construal of others and construal of the self in relation to others by measuring the conceptual distance between the *actual self*, *ideal self*, and *non-self* elements. Greater conceptual distance between the *actual self* and *non-self* elements implied greater perceived dissimilarity between the *self* and *others* [e.g (30)].

##### 3.4.3.1 Construal of others

In general, people with mental health conditions were said to idealise significant others (20), implying that they tended to construe other people positively. However, the same pattern of construing was observed for people with no identified mental health condition (20) and therefore may suggest that construing others positively was not unique to the experience of mental ill health. The RGTs completed by people diagnosed with schizophrenia substantiated the notion that people experiencing mental health conditions construed other people positively. Indeed, prior to achieving psychological stabilisation in schizophrenia, a person was said to construe *others* positively in comparison to the *self* (78). Moreover, *others*, and most strongly the essence of a *persecutory delusion*, tended to be construed as more malevolent and omnipotent than the *actual self* (22).

An exception to this pattern was revealed in the instance of depression. Construing others negatively was more evident in people diagnosed with depression, irrespective of the person having a comorbid diagnosis of PD, compared to people with no identified mental health condition, as illustrated in a comparison group study (69). This observation was upheld by a single case study of recurrent depression, which found that the *self* was construed as more similar to *others* they valued, and a *person without depression* as more similar to *others they disliked* (21). However, as per the findings of Rowe (21) discussed above, generalising this latter finding to other experiences of depression must be done with caution (93). Overall, these findings suggested that depression was linked with construing other people negatively, specifically those without depression.

Some authors challenged the notion that people diagnosed with a mental health condition strictly construed others in a unidimensional manner, i.e., either positively or negatively. Tschuschke and Dies (82) tentatively suggested that construing *parental* elements either extremely negatively or over-idealised was associated with higher distress symptomology when experiencing a mental health condition. This finding suggested that the extremity of construing, instead of the direction of construing (i.e., positively or negatively), was linked to experiencing a greater severity of mental ill health.

De Bonis et al. (69) discovered a similar result in the experience of borderline PD and comorbid depression by finding that people diagnosed with borderline PD with depression tended to hold a contrasted view of *others* (i.e., construing them as “good” and “bad”). However, holding a contrasted view of *others* may be distinctive to borderline PD, as people with a solitary diagnosis of depression and people with no identified mental health condition could not be differentiated from one another by this pattern of construing (69).

##### 3.4.3.2 Construal of self in relation to others

Predominantly, people with mental health conditions construed the *self* as dissimilar to *others* (20, 30, 70, 71, 74, 79). This notion was observed in presentations of depression (20, 70, 71), schizophrenia (30, 79), and OCD (74, 75), when compared to people with no identified mental health condition.

This pattern of low identification with *others* in depression, i.e., greater perceived dissimilarity between *actual self* and *non-self* elements, was one of the most consistent findings discovered through the RGT (95). Indeed, people diagnosed with depression construed greater dissimilarity between the *self* and *others* in comparison to people with no identified mental health condition and people diagnosed with other mental health conditions including anxiety disorders, PD, and mania, as observed in comparison group studies (20, 30, 70, 71). Interestingly, the conceptual distance between *actual self* and *non-self* elements was observed to decrease as depression improved (70), implying a movement towards greater social identification as depression alleviated. Collectively, these findings substantiated the notion that people with depression were likely to construe the self as dissimilar to other people.

A contrasting pattern of how the self was construed in relation to others was observed in anxiety disorders and the experience of mania. People diagnosed with these conditions were found to construe greater similarity between the *actual self* and *non-self* elements, in comparison to other mental health conditions such as depression (20) and schizophrenia (30). People diagnosed with an anxiety disorder or mania were denoted to construe the self in relation to others in a similar way to people with no identified mental health condition (20, 30).

Finally, the studies could not reach a consensus on the construing of the self in relation to others in people diagnosed with PD. One comparison group study suggested that greater dissimilarity between the *actual self* and *non-self* elements was construed by participants with a PD (79), compared to people with no identified mental health condition, while another proposed that such a difference was not evident (20).

##### 3.4.3.3 Construing of specific non-self elements

Some of the literature explicitly commented on the construing of specific *non-self* elements. Given the scarcity of studies that reported on specific non-self elements, the following findings are highly exploratory. A greater dissimilarity between the *self* and *parental* elements was construed within depression (71) and OCD (74), when compared to people with no identified mental health condition. Again, the reverse pattern seemed to be the case for anxiety disorders. *Parental* elements, specifically the *mother*, were construed more similarly to the *self* by participants who continued to experience heightened anxiety symptomology, relative to their counterparts who presented with reduced anxiety (73). However, it is important to note that the *mother* was construed negatively in this instance. People experiencing affective and schizoaffective disorders, most specifically schizoaffective psychosis and unipolar mania, appeared to construe their *partner* as more similar to their *actual* and *ideal selves*, compared to people with no identified mental health condition (29).

#### 3.4.4 Cognitive structure and construing

From the RGT, the authors made conclusions concerning the structure of the conceptual system and construing across mental health conditions. As the reviewers’ ability to assess what appeared to be comparative concepts was threatened by inconsistent definitions across studies, this review compartmentalised structure and process as the cognitive complexity, and the organisation and articulation of a person’s cognitive system. Thus, for the purpose of this review, the definition of cognitive complexity was summarised as the proportion of total variance accounted for by the first principal component and categorised as “simple” or “complex”, with larger proportions indicative of greater simplicity (e.g., (30), p. 250). Typically, an eigenvalue greater than 70% is generally considered to be indicative of “tight” construing (19, 96, 97). To note, the term cognitive complexity has been redefined in recent RGT literature as interpersonal cognitive differentiation (98); however, it will be referred to as cognitive complexity in this review due to this being the terminology used by the included studies. Cognitive organisation was classified as either “loose” or “tight”, with an overly loose organisation occurring when excess construct units are present and an excessively tight organisation when very few construct units exist (e.g., (83), p. 693). Articulation was subcategorised into “non-articulated”, either “monolithic” or “segmented”, and “articulated” structures. A “monolithic” structure comprised a singular primary cluster of constructs, while a “segmented” structure encompassed two or more primary clusters, without linking constructs between the clusters. An “articulated” structure consisted of two or more primary clusters joined by linking constructs [e.g., (74, 75)].

##### 3.4.4.1 Cognitive complexity

The RGT revealed that cognitive complexity was considered “simple” in depression and anxiety disorders, most specifically OCD (20, 30, 74). Conversely, a tentative interpretation of study findings suggested people experiencing mania and schizophrenia possessed highly “complex” cognitive systems (30); however, the reader is urged to interpret the findings of Ashworth et al. (30) with caution given that this was a non-significant finding. Affirming this, Phillips (84) indicated that greater cognitive complexity was associated with schizophrenia and a higher presence of paranoia and that individuals with greater cognitive complexity were more inclined to present with abstract and disorganised thinking in a sample of people with a range of different mental health conditions. Furthermore, cognitive complexity was depicted as significantly “more complex” in mania and schizophrenia than in depression, as shown in a comparison group study (30). From these findings, it could be inferred that a certain degree of cognitive complexity may be characteristic of specific mental health conditions. As such, cognitive complexity could be used as a dimension in which presentations of depression, anxiety, and OCD could be differentiated from presentations of psychosis and related experiences.

However, cognitive complexity may not serve as an appropriate dimension for distinguishing between people with an identified mental health condition and those without. No significant difference in the degree of cognitive complexity was found between a person with an identified mental health condition and a person with no identified mental health condition (71). Looking explicitly at specific mental health diagnoses, including depression, mania, schizophrenia, anxiety, OCD, and PD (20, 30, 71, 75), findings showed that cognitive complexity did not differ significantly between these mental health diagnoses and people with no identified mental health condition. However, one contradictory observation shown by Millar (74) suggested that cognitive complexity was in fact “more simple” in broad anxiety disorders and people experiencing OCD when compared to people with no identified mental health condition.

##### 3.4.4.2 Cognitive organisation and articulation

Findings regarding the organisation and articulation of the conceptual structure suggested that people with depression, a PD, or an anxiety disorder tended to have a “tightly” organised, “non-articulated”, and predominantly “monolithic” structure (20, 30). Krauthauser et al. (81) substantiated the notion that a “monolithic” conceptual structure tended to be associated with an anxiety or depressive disorder. People experiencing an anxiety or depressive disorder were found to experience greater cognitive contradiction within their construct system, a phenomenon that coincided with a tendency to have a “monolithic” structure and a less differentiated cognitive system (81). Moreover, a “monolithic” conceptual structure, greater cognitive contradiction, and poor differentiation appeared to coincide with greater psychological “disturbance”, higher anxiety and depression, and longer hospitalisation, for participants experiencing an anxiety or depressive disorder (81).

Several studies demonstrated that OCD specifically was said to be characterised by a “non-articulated” conceptual structure, specifically “monolithic” in nature (74–76). However, when collating comparison group studies, a consensus could not be reached on whether people with OCD and people with no identified mental health condition could be differentiated from one another on a measure of articulation (74–76).

In contrast, Ashworth et al. (30) portrayed that the articulation of the cognitive structure of people experiencing mania and schizophrenia was “non-articulated” but primarily “segmented”. One perspective revealed in some RGT studies was that schizophrenia was characterised by a single degree of organisation, “low” organisation, which appeared compatible with a “loose” cognitive system (78, 80). Klion (80) stated that two facets were encompassed within conceptual disorganisation—low construct interrelation (i.e., the degree of relationship between constructs) and low construct integration (i.e., the degree to which superordinate construct structures are utilised to subsume unrelated subordinate constructs)—and were dimensions that people with and without schizophrenia could be differentiated on. Specifically, the negative symptomology of schizophrenia (i.e., irritability, psychoticism, and motor retardation) was said to be associated with characteristics befitting “loose”, or low, organisation (78).

Conversely, an alternative suggestion was that an extremely “tight” or extremely “loose” pattern of cognitive organisation was observed in schizophrenia and schizoaffective disorders (77) and associated with the greatest volume of thinking errors indicative of thought disorder (83). In fact, construing of the self in either form (i.e., “tight” or “loose”) appeared to become “less extreme” as psychological stabilisation occurred in schizophrenia (77). This specific finding implied that it may be the extremity of cognitive organisation and not necessarily the form of organisation that is meaningful. While these findings were summarised to describe the cognitive organisation across the broad experiences of schizophrenia and psychosis, Adelman (1998) reported on distinct differences in organisation in separate conditions in this category of disorders. Adelman (77) showed that “tight” construing was present more frequently in schizoaffective disorder and that “loose” construing was more common in schizophrenia.

The findings suggested that distinct profiles of organisation and articulation were specific to depression, anxiety, OCD, PD, psychosis, and related disorders. The question of whether these conceptual structures were present only when people were actively unwell or whether it was inherent to them was not specifically explored by the included studies. However, some observations in the cases of schizophrenia and related disorders may have assisted with disentangling this. Adelman (77) observed that as psychological stabilisation occurred in schizophrenia, cognitive organisation became “less extreme”, i.e., less “loose” or less “tight”. Kubiak (78) noted that the resolution of a psychotic episode was linked with increased conceptual organisation. Therefore, both findings suggested that the profiles of conceptual systems denoted above may be specific to an active episode of schizophrenia and related disorders.

##### 3.4.4.3 Clinical presentation

In depression and anxiety disorders, including OCD, cognitive complexity was described as “simple”, and conceptual structure was predominantly labelled as “non-articulated”, specifically “monolithic”, and construed as “tight” (20, 30, 74–76). A cognitive system comprised of such facets was said to result in a person with a rigid, inflexible, single-dimensional view of the world (95). His/her behaviour was said to be organised along two construct poles on a single dimension, which can produce extreme behavioural consistency. However, whether this conceptual structure was unique to these identified mental health diagnoses remains in dispute (20, 30, 74, 75).

For schizophrenia spectrum and psychotic disorders, two predominant suggestions relating to conceptual structure and organisation emerged. Firstly, some authors described the conceptual system consistent with these groups of disorders as mainly highly “cognitively complex”, “non-articulated”, “segmented”, and extremely “loose” (20, 78, 80, 84). Collectively, this description illustrates a person who likely holds a disorganised, fragmented, unstable, and chaotic view of the self and the world. Construing was portrayed as incoherent, and the person was likely to experience confusion, excessive doubt, and uncertainty. This group of people was said to have an increasingly diverse repertoire of how to perceive the self as well as behaviourally respond (78). Conversely, the alternative was that the conceptual structure associated with schizophrenia spectrum and psychotic disorders was not restricted to a singular dimension of cognitive organisation. Indeed, in the experience of schizophrenia spectrum and psychotic disorders, both patterns denoted above were said to occur depending on whether the person was presenting with an extremely “loose” or exceptionally “tight” conceptual structure (77, 83).

## 4 Discussion

In a first of its kind, this systematic review of 21 studies synthesised findings relating to the content of construal, conceptual structure, and construing, captured through RGT methodology, completed by people whose diagnosed mental health condition necessitated inpatient psychiatric admission. A comprehensive exploration of the review’s aims was possible through the synthesis, leading to the emergence of several meaningful findings across a range of mental health diagnoses. As this review provided insight into the construal and construing processes that a person experiencing a mental health condition may use to make sense of the self, others, and their world, this synthesis may have the potential to significantly enhance therapeutic provision by highlighting potentially meaningful concepts that clinicians could use to better inform psychological assessment, formulation, and treatment.

One main finding that low self-esteem appeared to be present across a range of mental health conditions (20, 29, 30, 69–75, 78) was congruent with other literature that has demonstrated an association between low self-esteem and mental health conditions using alternative approaches to the RGT (99). This finding upheld the notions laid out by self-discrepancy theory that greater *actual*–*ideal* discrepancy (i.e., low self-esteem) results in emotional distress across diagnoses (31, 34). Therefore, low self-esteem is likely to be a central facet for exploration for any person accessing mental health services with an identified psychiatric diagnosis. It should be noted that the discrepancy between the actual self and ideal self is not the only way to explore self-esteem because there is also the distress associated with this discrepancy. However, RGT does not examine this distress dimension.

The synthesis of data pertaining to conceptual structure and construing revealed novel findings: mental health conditions possessed distinct profiles of cognitive complexity, organisation, and articulation, and people with an identified mental health condition tended to occupy a position of extremity on each dimension. Cognitive complexity emerged as a dimension potentially capable of distinguishing between presentations of depression, anxiety, PD, and OCD from presentations of psychosis and related disorders (30). Regarding conceptual organisation, Klion’s (80) and Kubiak’s (78) insights relating to construing in schizophrenia spectrum and psychotic disorders implied that these presentations could be distinguished from presentations of depression, anxiety, OCD, and PD based on this dimension. However, considering Adelman’s (74) and Phillips’ (83) notions of construing in schizophrenia spectrum and psychotic disorders, it implied that all aforementioned mental health conditions have the possibility to share the same form of cognitive organisation (i.e., an extremely “tight” conceptual structure). Therefore, the latter findings suggested that using a measure of organisation to differentiate between schizophrenia spectrum and psychotic disorders and presentations of depression, anxiety, OCD, and PD may not be possible.

Another important finding was that people with a mental health condition could not be consistently differentiated from people with no identified mental health condition on dimensions of cognitive complexity, organisation, and articulation (20, 30, 71, 74, 75). This finding, in conjunction with Adelman’s (77) and Kubiak’s (78) findings that psychological stabilisation coincided with the movement towards a more “moderate” conceptual organisation, could infer that to experience healthy, adaptive mental health, a person likely would fall in neither extreme and would exhibit “moderate” degrees of complexity, organisation, and articulation.

### 4.1 Clinical implications

The RGT findings that provided insight into the typical conceptual complexity, organisation, and articulation associated with certain mental health conditions could be useful for practicing clinicians to be aware of and provide areas of focus for psychological intervention. Some authors characterised schizophrenia and related disorders by “low” organisation and “loose” construing (78, 80), implying that increasing conceptual organisation could be conducive to psychological stabilisation, and this could be achieved through psychological therapy. However, to the authors’ knowledge, there are no studies that explored the causal role of increasing conceptual organisation through psychological therapy in achieving psychological stabilisation in schizophrenia and related disorders. Alternatively, important insights provided by Adelman (77) and Phillips (83) suggested that, in fact, a movement towards a more “moderate” conceptual organisation is what is necessary to achieve psychological stability. Without the latter insights, clinicians could be at risk of increasing cognitive organisation in an already extremely “tight” and “highly” organised conceptual system, which could have detrimental effects on psychological wellbeing. These specific findings stress the importance of clinicians identifying the direction of construing specifically displayed by their inpatient service users diagnosed with schizophrenia and related disorders. In doing so, clinicians may be more likely to provide meaningful therapeutic interventions that would hopefully support achieving a timely discharge and appropriate transition to community mental health care.

Regarding the construing of others, the “extremity” of construing (e.g., moderate and extreme) was observed to be as important, if not more important, than how others were construed (e.g., negatively or positively), especially for participants diagnosed with PD and comorbid anxiety or depressive symptomology (69, 82). Hence, the extremity to which a service user construes others either positively or negatively, in addition to how they construe others, should be a target for intervention when working therapeutically with inpatient servicer users diagnosed with PD.

Moreover, this review highlighted that certain relationships hold greater significance than others in some mental health conditions, for example, partner figures in affective and schizoaffective disorders (29) and parental figures in depression (71), anxiety (73), and OCD (74). Clinicians should therefore assess how a service user construes and relates to specific significant others (e.g., the conceptual distance between *self* and *non-self* elements) as part of psychological therapy with people with a specific mental health diagnosis who are receiving support in inpatient facilities. Following assessment, service users could be supported to develop more adaptive, helpful ways of construing these significant *non-self* elements, resulting in psychological benefits, which would ultimately support achieving hospital discharge.

Finally, the findings relating to low self-esteem occurring in participants with mental health conditions not only provided substantiating evidence for the link between self-esteem and mental ill health but also demonstrated the usefulness of the RGT as an assessment tool of self-esteem. Thereby, RGT could provide an alternative, idiosyncratic way of exploring self-esteem for clinicians who are working therapeutically with inpatient service users.

### 4.2 Strengths, limitations, and future research

Studies included in this systematic review varied in their application of the RGT, from the elements used, to the single or repeated application of the grid, to the method of construct elicitation. A strength of the RGT is its flexibility in application while maintaining the capability to reveal an individual’s conceptual system (15). However, several important limitations of the included literature were observed. Firstly, it became clear during the synthesis process that several definitions relating to the conceptual structure and construing had been used interchangeably across the literature, which meant that drawing conclusions about distinct concepts of cognitive complexity, articulation, and organisation was challenging. Nevertheless, by reflecting on the concepts collectively, many of the terms appeared compatible, and, subsequently, an overall picture of construing and conceptual structure within different mental health conditions appeared to emerge. However, readers are still advised to interpret these findings cautiously. Secondly, studies that requested participants to identify the preferred end of the construct pole often did not clearly define what was specifically meant by “preferred”. This lack of specificity could have meant there were varying interpretations of “preference” across studies, resulting in potential inconsistencies in collected data. A further disadvantage of requesting a participant to identify the preferred end is that a participant may experience ambivalence about which pole is preferred, which may threaten the validity of what is being measured (100).

Concerns arose relating to the studies that had used a “so-called” control group, often for comparative purposes. As mentioned above, as RGT originated from PCT, a classically personal, subjective approach (15), meaning RGT is characteristically idiosyncratic in nature (27, 101), drawing direct comparisons between individual repertory grids would not be viewed as typical, as a control group is normally used to compare one group to another on the same measure. The comparison group studies included in this review did not appear to ask participants directly to construe the self in relation to other people with different mental health conditions or persons without a diagnosis altogether; however, results appeared to be interpreted and presented in this way. While comparison group studies enabled some insights to be revealed, future studies may value including specific elements, such as “person without a mental health condition” or “person with other [specify] mental health condition”, to assess this.

Furthermore, despite this systematic review including information on 760 participants, from four different countries, spanning 45 years of research, the included studies were limited to western populations. This is an important limitation to highlight because a person’s self-construal can be shaped by his/her culture, and differences in self-construal between western and eastern cultures have been noted by some researchers (e.g., 139). Markus and Kitayama (102) emphasised an increased sense of interdependence in self-construal in eastern cultures, suggesting the self is construed as more interconnected with significant others, which differed to western cultures, in which independence and viewing the self as “separate” from others are accentuated. As the review findings could be culturally bound, to increase the generalisability of findings beyond western cultures, inclusion of studies from other geographical regions and cultures may be necessary. This limitation may be in part due to an actual scarcity of this type of research conducted with samples in non-western settings; however, it could also be due to biases introduced by the systematic review process itself. Due to the scope of the review, included studies were limited to publication in the English language; thus, instances of publication, location, and selection biases are possible. Restricting inclusion in this way may have prevented potentially meaningful studies written in different languages from being included (103). Indeed, eight studies were not considered for inclusion because the full text was not available in English. Thus, to expand the review’s breadth, future reviews could incorporate studies of multiple languages.

Eligible studies were limited to those conducted with inpatient participant samples because this approach increased the likelihood that the construals, construing, and conceptual structure were explored when a person’s mental health condition was more prominent or “active”, thereby better isolating the factor under investigation (i.e., mental ill health). Furthermore, limiting eligibility to inpatient participant samples ensured that the findings were specific to this population, which enabled more targeted clinical implications to be drawn. This specificity was essential in potentially enhancing the effectiveness of the psychologically driven assessment and intervention implications recommended by this review for those working with inpatient service users. Restricting the criteria in this way may have prevented meaningful insights from studies undertaken with outpatient samples from being collated as well as the opportunity to highlight meaningful similarities and/or differences between outpatient and inpatient participant samples. Therefore, to substantiate and potentially expand on the findings of this review, a future review encompassing inpatient- and outpatient-based participant samples may be valuable.

The use of an independent reviewer during the quality appraisal of included studies promoted objectivity and transparency regarding the methodological quality of included studies. As all studies were judged to contribute to the review and only three of the 21 studies obtained a moderate quality rating [i.e., (21, 72, 76)], no study was excluded based on methodological rigour. However, the quality appraisal process noted that almost all studies lacked sufficient information on recruitment data, did not critically discuss strengths and limitations, and fell short regarding the representativeness of their sample size. Therefore, because of these quality appraisal ratings, there are possible limitations to the conclusions produced by this review given that the conclusions are based on studies with potentially significant biases. The use of an independent reviewer for the systematic screening of articles enhanced consistency concerning article eligibility and strove to counteract subjectivity and certain biases that could have been introduced. Moreover, in accordance with the inclusion criteria, this review accepted grey literature, and therefore, the risk of publication biases was reduced.

### 4.3 Conclusions

This systematic review was the first to synthesise the findings of RGT studies that explored the content of construal, conceptual structure, and construing shown by people with an identified mental health condition necessitating inpatient admission. Similarities and differences in how a person perceived the self, others, and the self in relation to other people; the degree of cognitive complexity; and the organisation and articulation of conceptual structure were observed across different mental health conditions and between people with and without an identified mental health diagnosis. The review’s findings emphasise that clinicians should curiously assess and integrate the knowledge of conceptual systems into service user formulations to enable more targeted and meaningful psychological treatment. Clinicians should be cautious when service users present with a highly “tight” or extremely “loose” conceptual structure and instead strive to support the movement towards more “moderate” construing.
